# Shape based kinetic outlier detection in real-time PCR

**DOI:** 10.1186/1471-2105-11-186

**Published:** 2010-04-12

**Authors:** Davide Sisti, Michele Guescini, Marco BL Rocchi, Pasquale Tibollo, Mario D'Atri, Vilberto Stocchi

**Affiliations:** 1 Dipartimento DiSUAN, Sezione di Biomatematica, Università degli Studi di Urbino "Carlo Bo", Campus Scientifico Sogesta; Località Crocicchia - 61029 Urbino, Italy; 2 Dipartimento di Scienze Biomolecolari, Sezione di Ricerca sull'Attività Motoria e della Salute, Università degli Studi di Urbino "Carlo Bo", Via I Maggetti, 26/2 - 61029 Urbino, Italy

## Abstract

**Background:**

Real-time PCR has recently become the technique of choice for absolute and relative nucleic acid quantification. The gold standard quantification method in real-time PCR assumes that the compared samples have similar PCR efficiency. However, many factors present in biological samples affect PCR kinetic, confounding quantification analysis. In this work we propose a new strategy to detect outlier samples, called SOD.

**Results:**

Richards function was fitted on fluorescence readings to parameterize the amplification curves. There was not a significant correlation between calculated amplification parameters (plateau, slope and y-coordinate of the inflection point) and the Log of input DNA demonstrating that this approach can be used to achieve a "fingerprint" for each amplification curve. To identify the outlier runs, the calculated parameters of each unknown sample were compared to those of the standard samples. When a significant underestimation of starting DNA molecules was found, due to the presence of biological inhibitors such as tannic acid, IgG or quercitin, SOD efficiently marked these amplification profiles as outliers. SOD was subsequently compared with KOD, the current approach based on PCR efficiency estimation. The data obtained showed that SOD was more sensitive than KOD, whereas SOD and KOD were equally specific.

**Conclusion:**

Our results demonstrated, for the first time, that outlier detection can be based on amplification shape instead of PCR efficiency. SOD represents an improvement in real-time PCR analysis because it decreases the variance of data thus increasing the reliability of quantification.

## Background

In the last few years, real-time quantitative polymerase chain reaction (real-time PCR) has become the technique of choice for absolute or relative quantification of gene expression due to its rapidity, accuracy and sensitivity [1-3]. Furthermore, recent advances in the sequencing of the human genome, mRNA and miRNA expression profiling of numerous cancer types, disease-associated polymorphism identification and the expanding availability of genomic sequence information for human pathogens have led to marked growth in molecular diagnostics [4-6].

The gold standard quantification method (Ct method) in real-time PCR assumes that the compared samples have similar PCR efficiencies. However, quantification by real-time PCR is very sensitive to slight differences in PCR efficiencies among samples. Indeed, a small difference of 5% in PCR efficiency will result in a three-fold difference in the amount of DNA after 25 cycles of exponential amplification. Many factors present in samples as well as co-extracted contaminants can inhibit PCR, confounding template amplification and analysis [7-10]. This is a major problem when working with biological samples. Severe inhibition will lead to false-negative results, whereas a slight to moderate inhibition can result in an underestimation of the affected sample's DNA concentration [11]. Furthermore, amplification efficiency can fluctuate as a function of non-optimal assay design, enzyme instability, or the presence of inhibitors [12]. Although a variety of methods have been developed to quantify template DNA [11,13-17], very few allow simultaneous evaluation of template quantity and quality without the addition of an internal positive control that is co-amplified with the target of interest. Hence Bar and co-workers proposed a method (called KOD) based on amplification efficiency calculation for the early detection of non-optimal assay conditions [18,19]. This approach is extremely straightforward and effective, but it is based on a PCR amplification efficiency calculation for which there is still not a method fully accepted by the scientific community. A large number of studies have attempted to calculate amplification efficiency assuming that PCR is inherently exponential in nature. Based on the assumption of the log-linearity region, constant amplification efficiency is calculated from the slope of linear regression in that window [20-23]. An alternative approach is based on the observation that PCR trajectory can be effectively modelled by the sigmoid function [14,24] allowing PCR efficiency to be estimated using non-linear regression fitting [15,25,26]. Recently, a simplified approach called "linear regression of efficiency" has allowed us to estimate amplification efficiency by applying linear regression analysis to the fluorescence readings within the central region of amplification profile [27]. Notably, it has been demonstrated that estimates of PCR efficiency vary widely according to the approach that has been adopted [28].

Very recently, Tichopad et al. [29] introduced a new quality control test for quantitative PCR; in this procedure the first derivative maximum and the second derivative maximum were estimated using a logistic fitting on the PCR trajectory. This approach allowed them to monitor the first half of the curve using two parameters.

Our study aims to develop a quality test tool, which is not based on amplification efficiency estimation, in order to detect samples that do not show an amplification kinetic similar to those of standard samples. In this work, a non-linear fitting of Richards equation was used to parameterize PCR amplification profiles from a large sample set. The subsequent calculation of the variance of the estimated parameters and the development of a statistical measure based on the Mahalanobis distance allowed us to develop the SOD method (Shape based kinetic Outlier Detection). The SOD analysis of inhibited amplifications and the comparison of this method with KOD were investigated in detail.

## Methods

### Quantitative Real-Time PCR

The DNA standard consisted of a pGEM-T (Promega) plasmid containing a 104 bp fragment of the mitochondrial gene NADH dehydrogenase 1 (MT-ND1) as insert. This DNA fragment was produced by the ND1/ND2 primer pair (forward ND1: 5'-ACGCCATAAAACTCTTCACCAAAG-3' and reverse ND2: 5'-TAGTAGAAGAGCGATGGTGAGAGCTA-3'). This plasmid was purified using the Plasmid Midi Kit (Qiagen) according to the manufacturer's instructions. The final concentration of the standard plasmid was estimated spectophotometrically by averaging three replicate A_260 _absorbance determinations.

Real-time PCR amplifications were conducted using LightCycler^® ^480 SYBR Green I Master (Roche) according to the manufacturer's instructions, with 500 nM primers and a variable amount of DNA standard in a 20 *μ*l final reaction volume. Thermocycling was conducted using a LightCycler^® ^480 (Roche) initiated by a 10 min incubation at 95°C, followed by 40 cycles (95°C for 5 s; 60°C for 5 s; 72°C for 20 s) with a single fluorescent reading taken at the end of each cycle. Each reaction combination, namely starting DNA and inhibitor agent, was performed in triplicate and repeated in two separate amplification runs. All the runs were completed with a melt curve analysis to confirm the specificity of amplification and lack of primer dimers. *Ct *(fit point method) was determined by the LightCycler^® ^480 software version 1.2 and exported into an MS Excel data sheet (Microsoft) for analysis after background subtraction (available as Additional file 1). For *Ct *(fit point method) evaluation, a fluorescence threshold manually set to 0.4 was used for all runs.

### Estimation of PCR efficiency

The raw PCR data were used to calculate amplification efficiency. The PCR efficiency for each individual sample was derived from the slope of the regression line in the window of linearity [20]. Baseline correction and window of linearity identification were carried out using the latest version of LinRegPCR (v11.0) [23]. PCR efficiencies were estimated from four sample sets: standard amplification curves, standard amplification curves with the addition of tannic acid read-outs, standard amplification curves with the addition of IgG read-outs and standard amplification curves with the addition of quercitin read-outs. The window of linearity calculated from all the data sets encompassed the fluorescence threshold of 0.4 chosen for the quantitative analysis.

### Mathematical model of KOD

The mathematical model of KOD, based on efficiency, was proposed by Bar et al. [18]. Briefly, this was done comparing PCR efficiency of a sample (*x*_*eff*_) with the efficiencies of standard curve samples. A test sample is classified as an outlier if |*z*| > 1.96 with , where *μ*_*eff *_is the efficiency mean and *σ*_*eff *_is the standard deviation of the efficiency of standard curve samples. Alternatively, it is to be considered that the statistic  is distributed as a *χ*^2 ^with one degree of freedom; if *χ*^2 ^> 3.84, we can reject the null hypothesis at *α *= 0.05.

### Mathematical model of SOD

Shape based kinetic outlier detection (SOD) was based on the shapes of the amplification curves. In order to fit fluorescence raw data, nonlinear regression fitting of 5-parameter Richards function, an extension of the logistic growth curve, was used [11,25].(1)

where *x *is the cycle number, *F*_*x *_is the reaction fluorescence at cycle *x*, *F*_*max *_is the maximal reaction fluorescence, *F*_*b *_is the background reaction fluorescence and *b*, *c *and *d *represents the estimated coefficients. Nonlinear regressions for 5-parameter Richards functions were performed determining unweighted least squares estimates of parameters using the Levenberg-Marquardt method.

The shape parameters used were the plateau value of amplification curve (*F*_*max*_), tangent straight line slope in inflection point (*m*) and y-coordinate of inflection point (*Y*_*f*_) (Additional file 2).

The y-coordinate of inflection point (*Y*_*f*_) was calculated as follows:(2)

and the tangent straight line slope (*m*) was estimated as:(3)

Normal distribution of *F*_*max*_, *Y*_*f *_and *m *parameters, obtained from standard samples, was checked using the Kolmogorov-Smirnov test for normality; the significance of the correlation between these parameters and input DNA concentrations, expressed as *Log(DNA)*, was tested with a *t *test as follows:(4)

where *r *is the Pearson coefficient and *n *the sample size (*n *= 72). The multivariate normality of the adopted reference set was evaluated according to Rencher AC [30] (Additional file 3). In addition, the asymmetry (*Asym*) of the amplification curves was estimated as follows:(5)

replacing *Y*_*f *_and *F*_*max*_, Eq. 5 can be simplified as: . In agreement with this equation the curve is symmetric (that is Asym = 0) when d = 1, or 2*Yf = Fmax. On the contrary, when d>1 we have 2*Yf<Fmax (the curve is asymmetric) hence Asym>0.

### Statistical model of SOD

After developing a method to estimate three different shape-parameters (*F*_*max*_, *Y*_*f*_, *m*), the next step was to set a criterion to identify test samples that deviated from expected values. This was done using sample vector  which can be calculated for each experimental amplification; if ***y ***belongs to a multivariate normal distribution, with mean vector  and **Σ **the corresponding variance-covariance matrix, the ***(y-μ)'***Σ**^-1^*(y-μ) ***value (Mahalanobis distance) has asymptotic *χ*^2 ^distribution, with 3 degrees of freedom. The Mahalanobis distance is based on correlations between variables through which different patterns can be identified and analyzed. It is a useful way of determining the similarity of an unknown multivariate sample set to a known one. It takes into account the correlations of the data set and is not dependent on the scale of measurements. Mean vector and variance-covariance matrix were calculated from shape parameters of standard curve samples. Then if *χ*^2 ^> 7.81, we can reject the null hypothesis (with *α *= 0.05) and establish that the shape of the amplification curve is different from the shape of the standard curve samples, considering all three parameters [30]. All elaborations and graphics were obtained using Excel (Microsoft), Statistica 6.0 (Statsoft) and Statistical Package for Social Sciences (SPSS 13.0).

## Results

### Standard curve SOD analysis

The SOD model relies on the assumption that in order to achieve a reliable quantification, the amplification curves of unknown samples should not be significantly different from those of the standard curve. We introduced the idea that the amplification kinetic can be monitored by the shape of the amplification curve. The shape of amplification curves was parameterized using the nonlinear regression fitting of the Richards function on the fluorescence readings [11]. This mathematical procedure allowed us to obtain the five parameters characteristic of the Richards equation. These values were subsequently used to calculate the slope of the tangent at the inflection point (*m*), the y-coordinate of the inflection point (*y*_*f*_) and the maximum fluorescence value (*F*_*max*_) of the reading. Finally, these three parameters allowed us to create a "fingerprint" for each amplification curve.

**Figure 1:**
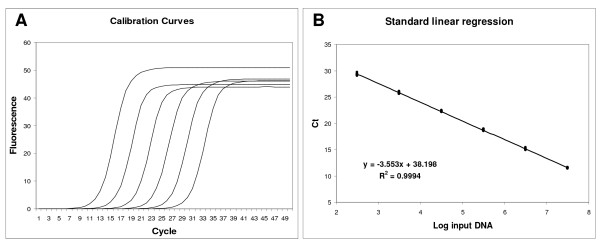
**Linear regression analysis of standard samples**. The amplification profiles were produced by averaging the fluorescence readings of twelve replicate reactions (A). Linear regression obtained plotting Log input DNA versus *Ct *(B).

**Figure 2:**
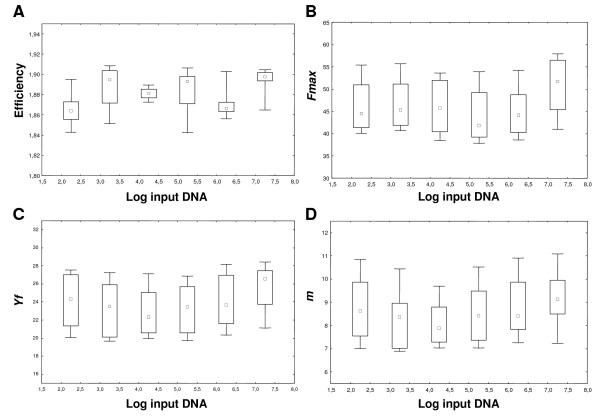
**Efficiency and shape parameter values of standard curve samples**. The plots of efficiency (A), *Fmax *(B), *Yf *(C) and *m *(D) were shown; we reported in abscisse the Log transformation of input DNA and in ordinate the parameter value. The square represents the median, the length of the box shows the interquartile range and the whiskers indicate the min-max values of the estimated parameters.

Based on this assumption, the parameters *m*, *y*_*f *_and *F*_*max *_of the amplifications used to build a standard curve should not be significantly different from one another and should not be correlated with input DNA. To verify this assumption, a standard curve was generated over a wide range of input DNA (3.14 × 10^7^-3.14 × 10^2^; Fig. 1; Additional files 1). Table 1 shows the mean, SD, and Kolmogorov-Smirnov test from a total of 72 runs. These results demonstrated that *m*, *y*_*f *_and *F*_*max *_were normally distributed, even though they showed a different dispersion. Subsequently, the relationship between *m*, *y*_*f *_and *F*_*max *_and the Log of the starting DNA template was studied. As shown in Fig. 2, there was not a significant correlation between the Log of input DNA and these parameters (*F*_*max*_: *R*^2 ^= 0.017 *p *= 0.28; *y*_*f*_: *R*^2 ^= 0.033 *p *= 0.12; *m*: *R*^2 ^= 0.030 *p *= 0.14). In fact, determination coefficients *(R*^2^*) *quantified only a very low proportion of parameter variances less than 3,3%.

**Table 1:** One-Sample Kolmogorov-Smirnov test of calibration curve.

	*Eff*	*y*_*f*_	*m*	*Fmax*
N = 72				
Mean (S.D.)	1.88 (0.02)	23.89 (2.86)	8.61 (1.20)	46.41 (6.07)
K-S value (*p*)	0.99 (0.28)	1.25 (0.09)	0.75 (0.63)	1.13 (0.15)

Means (standard deviation) and Kolmogorov-Smirnov (K-S) test value (probability) of the following parameters: LineReg efficiency (*Eff*), ordinate value of inflection point (*y*_*f*_), slope of tangent straight line in inflection point (*m*) and plateau value (*Fmax*).

In order to objectively define an amplification profile as an outlier, we introduced the variable Log(*N*_*ob*_/*N*_*exp*_), which estimates errors from quantification analysis using the *Ct *method. This variable relies on the residues estimated as the difference between calculated molecules, using the *Ct *method (Log of Number of Observed Molecules, referred to as Log*N*_*ob*_), and input DNA molecules (Log of Expected Molecules, referred to as Log*N*_*exp*_; in fact Log*N*_*ob*_-Log*N*_*exp *_= Log(*N*_*ob*_/*N*_*exp*_)). The ratio Log(*N*_*ob*_/*N*_*exp*_) showed a normal distribution satisfying the assumption of homoscedasticity (Additional file 4). It is thus possible to determine a 95% confidence interval (CI) for the variable Log(*N*_*ob*_/*N*_*exp*_). These residues showed a normal distribution regardless of the starting DNA template, with the average equal to zero and the standard deviation constant (*σ *= 0.041). In our database, out of a total of 72 runs used to construct the standard curve, 6 runs showed the ratio Log*(N*_*ob*_/*N*_*exp*_*) *out of the CI (Additional file 5). Subsequently, PCR efficiency (*E*_*ff*_) was also estimated for each amplification curve; the LinRegPCR software [20,23] was used to fit the data points in the optimal range of the PCR exponential phase to obtain an automated evaluation of *E*_*ff *_(Table 1).

To determine how well outlier samples can be identified by KOD and SOD, we applied these statistical analyses to the runs of the standard curve; in particular we found that KOD identified 2 runs over the *χ*^2 ^threshold value of 3.84 while SOD revealed 3 runs out of the CI (Additional file 5). These outliers are probably false-positives due to the definition and intrinsic properties of the 95% CI.

### Inhibitor effects on real-time amplification

**Figure 3:**
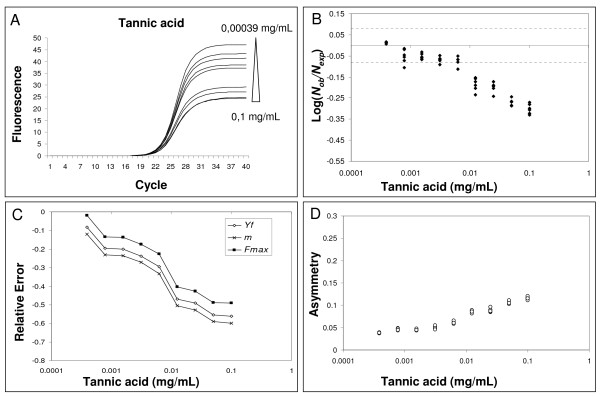
**Effect of tannic acid inhibition on amplification curve shape**. Left upper panel: amplification profiles obtained from samples with equal starting number of template molecules and increasing inhibitor concentrations. For each inhibitor concentration only an amplification curve was plotted (instead of all 6 replicates). Values over and under triangle indicator (at the upper right of the Fig.) show the lowest and the highest inhibitor concentration used (A). Right upper panel: effect of PCR inhibition on the ratio *Log(N*_*ob*_/*N*_*exp*_*) *in the presence of equal starting number of template molecules and increasing inhibitor concentration. The ratio *Log(N*_*ob*_/*N*_*exp*_*) *represents the residues obtained from linear regression of calibration curves where Log*N*_*ob *_is the number of calculated molecules using *Ct *method and *N*_*exp *_is the number of expected molecules. Each symbol represents a single run. The abscisse axis is the mean and the dotted lines are the 95% confidence interval of the *Log(N*_*ob*_/*N*_*exp*_*) *ratio calculated from standard curve runs (B). Left lower panel: variation of *F*_*max*_, *Y*_*f*_. and *m *versus increasing inhibitor concentration. The variation is expressed as Relative Error = ; where  is the mean of parameter calculated for each inhibitor concentration;  represents the mean of parameter value from standard curve samples (C). Right lower panel: asymmetry values versus increasing inhibitor concentration. Asymmetry was computed as the following ratio:  (D).

Tannic acid oxidizes to form quinones which covalently bind to *Taq *DNA polymerase inhibiting its activity [31]. Real-time amplification plots from 3.5 × 10^4 ^DNA molecules in the presence of increasing concentrations (0-0.1 mg per mL) of tannic acids were obtained. All the quantification values were obtained using the *Ct *method. The resulting amplification curves and the corresponding quantifications demonstrate the effects of inhibition on real-time analysis (Fig. 3A and 3B). As the tannic acid concentration increased, the *Ct *values went up steadily leading to an underestimation of the starting molecules. This quantification error was highlighted when Log(*N*_*ob*_/*N*_*exp*_) dropped out the corresponding CI (Fig. 3B). Suppressed amplification was demonstrated by the calculations of efficiency using LinRegPCR procedure (Additional file 5). The observed errors were the result of the progressive reduction of the plateau, linear phase length and slope of the inhibited curves; together these effects led to increasing *Ct *values (Fig. 3A) [19,32].

These data led us to investigate the modifications of the parameters *m*, *y*_*f *_and *F*_*max *_in response to increasing inhibitor concentrations. Fig. 3C shows the increase in relative error of *m*, *y*_*f *_and *F*_*max *_in the presence of increasing tannic acid concentrations. Notably, these results also showed that curve asymmetry (Eq. 5) increased with higher inhibitor concentrations. This in turn demonstrates that not only the slope (*m*) and plateau (*F*_*max*_) of the curve decreased but also the shape changed moving towards a more and more Richards' type kinetic (Fig. 3D).

**Figure 4:**
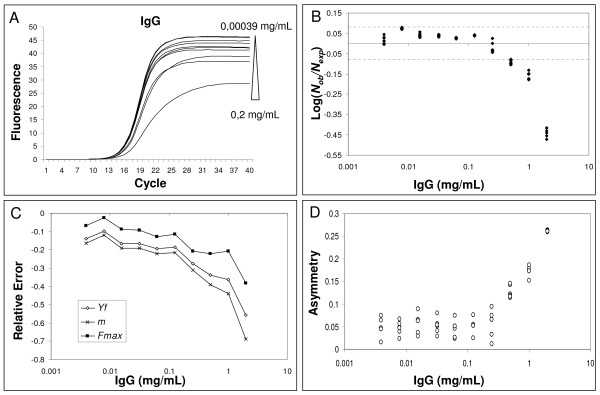
**Effect of IgG inhibition on amplification curve shape**. For details refer to figure legend 3.

**Figure 5:**
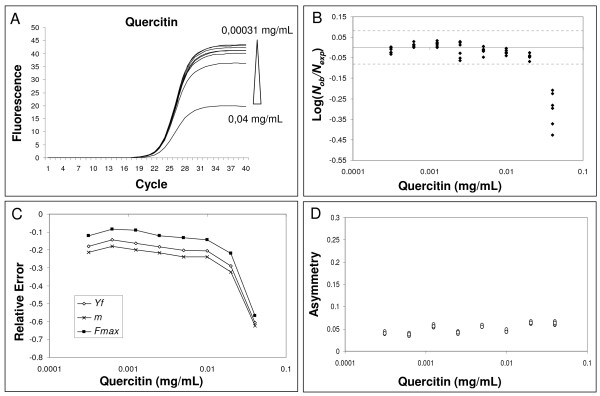
**Effect of quercitin inhibition on amplification curve shape**. For details refer to figure legend 3.

Subsequently, we evaluated the effects of IgG and quercitin, molecules known to inhibit PCR, on amplification kinetics [11,32,33]. Both these molecules result in a significant underestimation of starting DNA molecules at high inhibitor concentrations (Fig. 4B and 5B). As shown in Fig. 4 and 5, we always found a change in parameters *m*, *y*_*f *_and *F*_*max *_when the quantification error occurred.

Furthermore, the asymmetry analysis showed an interesting singularity in the quercitin effects compared to those of tannic acid and IgG. In fact, quercitin led to kinetic alterations without a significant effect on the curve symmetry (Fig. 5D).

### SOD versus KOD analysis

SOD and KOD analyses were used to identify samples with aberrant PCR kinetics, due to inhibitor presence, which might lead to erroneous quantifications. *F*_*max*_, *m *and *y*_*f *_values calculated from each amplification curve, obtained in the presence of increasing tannic acid, IgG or quercitin concentrations, were used to estimate the *χ*^2^_*SOD *_value. Hence if the *χ*^2^_*SOD *_value from an amplification curve was higher than the threshold value 7.81, the quantification was defined as an outlier. PCR efficiencies were also estimated and *χ*^2^_*KOD *_values determined from the same amplifications. Quantification curves with a *χ*^2^_*KOD *_values over 3.84 were rejected.

**Figure 6:**
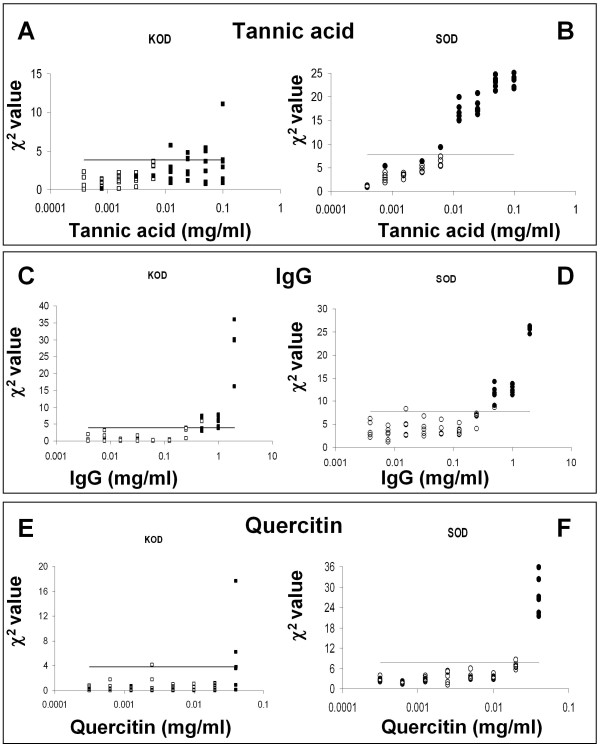
**Values of KOD and SOD related of each amplification curve versus *Log *of inhibitor concentration**. Symbols (squares and dots) represent the *χ*^2 ^values related to each amplification curve obtained in the presence of different inhibitor concentrations. The horizontal continuous lines are the critical values for detecting outliers (left panels: the KOD *χ*^2 ^critical value is 3.84; right panels: the SOD *χ*^2 ^critical value is 7.81; with *α *= 0.05). Different inhibitors were used: Tannic acid (A-B), IgG (C-D) and Quercitin (E-F). True outliers (represented by black symbols; squares for KOD and dots for SOD) are amplification curves with *Log(N*_*ob*_/*N*_*exp*_*) *ratio out of 95% confidence interval, while white symbols represent acceptable runs with *Log(N*_*ob*_/*N*_*exp*_*) *ratio included in 95% confidence interval. The 95% confidence interval has been obtained from the amplification curves of the standard samples. The black symbols, over the horizontal continuous line, are runs correctly detected as outliers. Conversely, black symbols under this line are undetected outliers.

Hence the SOD and KOD performances were evaluated according to their ability to identify an amplification as an outlier when the Log(*N*_*ob*_/*N*_*exp*_) ratio is not within 95% CI. The results obtained by SOD and KOD analyses in the presence of increasing tannic acid concentrations are shown in Fig. 6A and 6B. When tannic acid concentrations ranging from 0.1-0.0125 mg/mL were added, all the obtained curves had significant quantification errors (Fig. 6A and 6B; full symbols indicate samples that showed the ratio Log(*N*_*ob*_/*N*_*exp*_*) *below the lower limit of 95% CI). These curves were associated with *χ*^2^_*SOD *_values higher than the threshold value of 7.81 (Fig. 6B; the horizontal line shows *χ*^2^_*SOD *_threshold value). In this concentration range, KOD analysis appeared to be less powerful than SOD. In fact, KOD found as outliers (*χ*_*KOD*_^2 ^> 3.84) only 8 of the 24 curves showing a Log(*N*_*ob*_/*N*_*exp*_) ratio out of 95% CI (Fig. 6A). There were no outliers under 0.00625 mg/mL tannic acid concentration, with the exception of some amplifications that were randomly out of the CI.

SOD and KOD analyses were also applied to real-time quantifications in the presence of IgG or quercitin as inhibitors. When amplification reactions were conducted in the presence of 2-0.5 mg/mL IgG, the suppression of amplification was efficiently revealed by both SOD and KOD, though SOD was more sensitive than KOD. In fact, SOD highlighted 17 outliers versus 15 revealed by KOD out of a total of 17 outliers (in the presence of IgG 17 runs led to a Log(*N*_*ob*_/*N*_*exp*_) out of 95% CI) (Fig. 6C and 6D). Analogous results were also obtained for quercitin. In the presence of 0.04 mg/mL of quercitin, SOD found 6 outliers compared to the 3 revealed by KOD out of a total of 6 outliers (Fig. 6E and 6F; for details of SOD and KOD analysis see Additional file 5).

Finally, we defined as true positives (*TP*) those amplifications showing *χ*^2^>threshold value and those that led to a Log(*N*_*ob*_/*N*_*exp*_) ratio out of the 95% CI. Conversely, false positives (*FP*) were defined as samples that showed the *χ*^2^>threshold value and a Log(*N*_*ob*_/*N*_*exp*_) ratio within the 95% CI. Consequently, true negatives (*TP*) were those amplifications showing *χ*^2^<threshold value that led to a Log(*N*_*ob*_/*N*_*exp*_) ratio within the 95% CI and false negatives (*FN*) those showing *χ*^2^<threshold value and Log(*N*_*ob*_/*N*_*exp*_) ratio out of the 95% CI.

Based on these definitions, the *'sensitivity' *of SOD and KOD is represented by the ratio  while the *'specificity' *is the ratio: . Table 2 shows that SOD was more sensitive than KOD in all the tested settings, while SOD and KOD were equally specific in the presence of IgG and quercitin. SOD was also more specific than KOD in the presence of tannic acid.

**Table 2:** Sensitivity and specificity of KOD and SOD analysis.

KOD	Tannic Acid	IgG	Quercitin
	
Sensitivity	0.30***	0.76***	0.50***
Specificity	0.94	0.96	0.98
**SOD**	**Tannic Acid**	**IgG**	**Quercitin**
	
Sensitivity	0.93***	1.00***	1.00***
Specificity	1.00	0.94	0.98

The comparison between PCR amplification profiles in the presence of different inhibitors using KOD and SOD analyses showed that SOD is significantly more sensitive than KOD in all the tested conditions (*** p < 0.001; Z test for difference of proportion), whereas KOD and SOD specificity is similar in the same conditions (p > 0.05; Z test for difference of proportion).

## Discussion

A topic of great interest is the development of hand-free tools for the detection of aberrant amplification profiles in real-time PCR analysis. Real-time PCR has rapidly become the most widely used technique in nucleic acid quantification. Although real-time PCR analysis has gained considerable attention in many fields of molecular biology, it is still troubled by significant technical problems [34]. Hence the present study has focused on the investigation of a new outlier detection approach which is not based on the PCR efficiency estimate but rather on the shape of the amplification profile.

The amplification nature of PCR makes it vulnerable to small differences in efficiencies of compared samples [20]. In fact, the current "gold standard" in real-time PCR analysis, the threshold cycle method (called *Ct *method), requires similar PCR efficiencies among compared samples.

However, dissimilarity in PCR efficiency results from different starting material sources, for example, different types of tissues [9]. Such differences might also be found when inhibitors of *Taq *DNA polymerase are present in cDNA samples [35] or in the presence of low quality SYBR green and/or dNTPs [36,37]. Furthermore, the frequency of PCR inhibition [38] and different inhibitory effects even among replicates [39] highlight the need of kinetic quality assessment for each sample. Hence Bar et al. [18] proposed a statistical method, called KOD, to detect samples with dissimilar efficiencies.

KOD searches for outliers based on the main assumption that to obtain a reliable quantification, PCR runs have to show efficiencies which are not significantly different from each other. This condition is verified comparing the slopes of the straight-line regression calculated in the window-of-linearity after the log-transformation of each read-out fluorescence. In other words, if we return to raw data, the profile of the exponential curves in the window-of-linearity, mustn't be significantly different among compared runs. In the development of the SOD method we extended this concept to the whole curve, and all the runs included in the analysis have to show comparable amplification profiles.

The *Ct *method is based on the analysis of a serially diluted target. An example of this approach is presented in Fig. 1A careful examination of the obtained amplification profiles illustrates the central principle of the SOD method: all amplification curves are similar in shape and only the profile position is related to target quantity. The first amplification profiles, corresponding to the most concentrated samples, are found on the left, whereas samples with an increasing dilution factor regularly shift towards the right. This observation led us to the insight that an exclusion criterion could be based on the difference in shape rather than efficiency. This is in agreement with the work by Rutledge and Stewart [40] in which these authors described the amplification curve as a function of efficiency. Hence if efficiency determines the shape of a curve, by monitoring the shape of an amplification profile, information concerning the efficiency of amplification can be obtained.

Firstly, a "fingerprint" for each amplification curve using *m*, *y*_*f *_and *F*_*max *_resulting from the fitting of the Richards equation on raw data was obtained. Subsequently, these parameters were used to obtain the variance-covariance matrix in order to calculate the Mahalanobis distance [30]. This statistical measure is based on correlations among variables through which different patterns can be identified and analysed. In particular, the SOD analysis made use of the Mahalanobis distance to determine the similarity of an unknown sample compared to the standard set. This approach was very useful because it allowed us to evaluate not only the variance of single parameters (*m*, *y*_*f *_and *F*_*max*_), but also to quantify the reciprocal co-variations among *m*, *y*_*f *_and *F*_*max*_.

*F*_*max *_was considered in the development of SOD because this parameter demonstrates successful amplification and usually, in suboptimal amplification conditions, the read-outs do not reach characteristic *F*_*max *_values [9]. Examining our database, it was noted that *F*_*max *_showed high variance, thus it slightly affects *χ*^2^_*SOD *_alone, but *F*_*max *_had a significant impact on the variance-covariance matrix. The parameter *m *describes the slope of the curve in the inflection point [11]. In our model, the higher the value of *m*, the higher the amplification rate is. However, this estimator does not directly indicate the amplification efficiency understood as the proportion between current and previous product amounts [38]. Finally, the asymmetry of amplification profiles was monitored by the relationship between *F*_*max *_and *y*_*f*_. It has been demonstrated that absolutely symmetrical PCR curves seldom occur, justifying the introduction of a five-parameter fit [25]. Furthermore, in our previous work [11], it was demonstrated that the amplification reaction may deviate from a symmetric sigmoid curve to an asymmetric sigmoid (well described by Richards equation) in the presence of suboptimal efficiency. In fact, the goodness of fit of the logistic model progressively decreased with lower efficiency suggesting a change of PCR curve amplification shape [32].

The correlation analysis between *m*, *y*_*f *_and *F*_*max *_obtained from the standard curve and input DNA demonstrated that these shape parameters are concentration-independent. This supports our experimental hypothesis that all the amplification curves of the standard curve are similar in shape and only the profile position determines target quantity. In the presence of PCR inhibition, it was found that increasing concentrations of tannic acid and IgG resulted in decreasing *F*_*max *_and *m *values, while asymmetry increased with higher inhibitor concentrations (when asymmetry increases, *y*_*f *_decreases more than the corresponding *F*_*max*_; Fig. 3 and 4). It may be that tannic acid inhibition is simply due to fluorescence quenching since we found a dramatic decrease in *F*_*max *_and a slide curve slope decrease. However, we also showed that fluorescence asymmetry increased demonstrating that tannic acid produced an amplification kinetic distortion. The addition of quercitin to PCR amplifications produced very interesting data. In fact, we found decreased *F*_*max *_and *m *values in the presence of high inhibitor concentrations, however this flavonid did not induce an asymmetric modification of the curves (Fig. 5D). The reported data clearly demonstrate that the SOD method can identify non-optimal PCR kinetics resulting from different inhibition models. Furthermore, the results obtained in the presence of quercitin highlight the importance of using a multivariate approach.

When comparing SOD to KOD performance, it was found that SOD was more sensitive than KOD in all the tested settings. SOD and KOD were equally specific in the presence of IgG and quercitin, whereas SOD was more specific than KOD in the presence of tannic acid.

Furthermore, the SOD method presents several advantages over KOD; SOD is completely hand-free. Indeed, it is not necessary for the user to identify a window of analysis as in the KOD method, and more importantly, SOD does not rely on a constant efficiency value avoiding all the problems connected with its determination [28,40,41]. As previously reported, variable PCR efficiency determination can lead to different results contributing to erroneous and spread quantifications [19]. Moreover, log-transformation of fluorescence data that could be responsible for bias in the analysis are avoided.

The SOD method has been developed for the chemistry Sybr Green, and the application of this procedure to other chemistries such as TaqMan, needs to be evaluated extensively.

Very recently, Tichopad et al. [29] proposed a new KOD procedure based on Malahanobis statistic [30]. In this study the first derivative maximum and the second derivative maximum were estimated using a logistic fitting on the central portion of the PCR trajectory. Using these two parameters these authors proposed monitoring only the first half of the curve. On the contrary, the SOD method is based on the possibility of describing the whole PCR trajectory using Richards equation. SOD represents a continuation and an extension of the application of Richards equation to real-time PCR readings [11]. We think that the SOD method introduces original concepts that are not found in the recently developed method described by Tichopad et al. [29]. SOD takes advantage of the possibility of describing the shape of the whole PCR trajectory through the combination of the parameters *m*, *y*_*f *_and *F*_*max *_while the method by Tichopad et al. [29] focuses on two key points of the trajectory: the maximum of the first and second derivative. Furthermore, in the SOD method we used quite a different metric approach. Although other multivariate methods are available for similar tasks (support vector machines, K-means cluster), we used asymptotic distribution of the Mahalanobis distance because it is a logical extension of the KOD method, which is based on univariate normal distribution.

## Conclusion

We demonstrated for the first time that a comparison of the shape variation of an amplification profile with the shape of standard profiles can be used to exclude aberrant samples from *Ct *analysis. This allows us to avoid the spread of results and therefore increases the potential of quantification analysis.

Hence we propose SOD as a hand-free quality control method in real-time PCR analysis with applications in any field of molecular diagnostics.

## Abbreviations

Ct: threshold cycle; IgG: immunoglobulin G; SOD: shape based kinetic outlier detection; KOD: kinetic outlier detection; Asym: Asymmetry.

## Authors' contributions

MG and DS carried out the design of the study, participated in data analysis, developed the *SOD *method and drafted the manuscript. MBLR participated in data collection and analysis and critically revised the manuscript. PT carried out the real-time PCR. DM participated in data collection. VS participated in the design of the study and critically revised the manuscript. All authors read and approved the final manuscript.

## Supplementary Material

Additional file 1 **Fluorescence data and fitting elaboration of standard sample amplifications (standard curve) and amplifications obtained in the presence of: tannic acid, IgG and quercitin**. Click here for file

Additional file 2 **Analytical solutions for the y value of the inflection point *(Y*_*f*_.*) *and the slope of tangent straight-line (*m*) crossing the inflection point**. Click here for file

Additional file 3 **A) Chi-square distribution of the squared distances about the population mean vector (D2 = *(y-μ)'Σ*^-1^*(y-μ)*) with 3 degrees of freedom**. B) Scatter plots of all pairs of variables *F*_*max*_, *Y*_*f *_and *m*. Click here for file

Additional file 4 **P-P plot of the variable *Log(N*_*ob*_/*N*_*exp*_*)***. Click here for file

Additional file 5 **KOD and SOD elaborations of standard sample amplifications (standard curve) and amplifications obtained in the presence of: tannic acid, IgG and quercitin**. Click here for file
