# Supplementary material for: Shape based kinetic outlier detection in real-time PCR
Source: BMC Bioinformatics. 2010 Apr 12;11:186. doi: 10.1186/1471-2105-11-186 (PMC2873533; doi:10.1186/1471-2105-11-186)
Supplement: Additional file 2 — Analytical solutions for the y value of the inflection point (Yf.) and the slope of tangent straight-line (m) crossing the inflection point. [file 1471-2105-11-186-S2.DOC]

The y-coordinate of inflection point (*Y_f_*) was calculated as follows:

First derivative of Richards equation:

$$\frac{dF_{x}}{dx}=F_{\max}\left(-d\right)\left[1+e^{-\frac{1}{b}\left(x-c\right)}\right]^{-d-1}e^{-\frac{1}{b}\left(x-c\right)}\left(-\frac{1}{b}\right)$$

therefore:

$$\frac{dF_{x}}{dx}=\frac{dF_{\max}}{b}e^{-\frac{1}{b}\left(x-c\right)}\left[1+e^{-\frac{1}{b}\left(x-c\right)}\right]^{-d-1}$$

Second derivative of Richards equation:

$$\frac{d^{2}F_{x}}{dx^{2}}=\frac{dF_{\max}}{b}\left\{e^{-\frac{1}{b}\left(x-c\right)}\left(-\frac{1}{b}\right)\left(1+e^{-\frac{1}{b}\left(x-c\right)}\right)^{-d-1}+e^{-\frac{1}{b}\left(x-c\right)}\left(-d-1\right)\left(1+e^{-\frac{1}{b}\left(x-c\right)}\right)^{-d-2}e^{-\frac{1}{b}\left(x-c\right)}\left(-\frac{1}{b}\right)\right\}$$

therefore:

$$\frac{d^{2}F_{x}}{dx^{2}}=-\frac{dF_{\max}}{b^{2}}e^{-\frac{1}{b}\left(x-c\right)}\left[1+e^{-\frac{1}{b}\left(x-c\right)}\right]^{-d-2}\left[1+e^{-\frac{1}{b}\left(x-c\right)}-de^{-\frac{1}{b}\left(x-c\right)}-e^{-\frac{1}{b}\left(x-c\right)}\right]$$

and finally:

$$\frac{d^{2}F_{x}}{dx^{2}}=-\frac{dF_{\max}}{b^{2}}e^{-\frac{1}{b}\left(x-c\right)}\left[1+e^{-\frac{1}{b}\left(x-c\right)}\right]^{-d-2}\left[1-de^{-\frac{1}{b}\left(x-c\right)}\right]$$

Determination of abscisse of inflexion point (*x_flex_*):

$\frac{d^{2}F_{x}}{dx^{2}}=0$ when

$$1-de^{-\frac{1}{b}\left(x-c\right)}=0$$

next:

$$x_{flex}=c+b\ln d$$

**Determination of ordinate in *x_flex_* (*Y_f_*)**

$$Y_{f}=\frac{F_{\max}}{\left[1+e^{-\frac{1}{b}\left(c+b\ln d-c\right)}\right]^{d}}$$

therefore:

$$Y_{f}=\frac{F_{\max}}{\left[1+e^{\ln d^{-1}}\right]^{d}}$$

$$Y_{f}=\frac{F_{\max}}{\left(1+\frac{1}{d}\right)^{d}}$$

and finally:

$Y_{f}=F_{\max}\left(\frac{d}{d+1}\right)^{d}$ (Eq. 2 of the text)

Determination of the slope of tangent straight-line (*m*) crossing the inflection point:

*m* =$F^{'}x_{flex}=\frac{dF_{\max}}{b}e^{-\frac{1}{b}\left(c+b\ln d-c\right)}\left[1+e^{-\frac{1}{b}\left(c+b\ln d-c\right)}\right]^{-d-1}$

$$F^{'}x_{flex}=\frac{dF_{\max}}{b}\left(\frac{1}{d}\right)\left[1+\frac{1}{d}\right]^{-d-1}$$

therefore:

*m* =$F^{'}x_{flex}=\frac{F_{\max}}{b}\left[\frac{d}{d+1}\right]^{d+1}$ (Eq. 3 of the text)
