# Supplementary material for: Shape based kinetic outlier detection in real-time PCR
Source: BMC Bioinformatics. 2010 Apr 12;11:186. doi: 10.1186/1471-2105-11-186 (PMC2873533; doi:10.1186/1471-2105-11-186)
Supplement: Additional file 3 — A) Chi-square distribution of the squared distances about the population mean vector (D2 = (y-μ)'Σ-1(y-μ)) with 3 degrees of freedom. B) Scatter plots of all pairs of variables Fmax, Yf and m. [file 1471-2105-11-186-S3.PPT]

## Slide 1
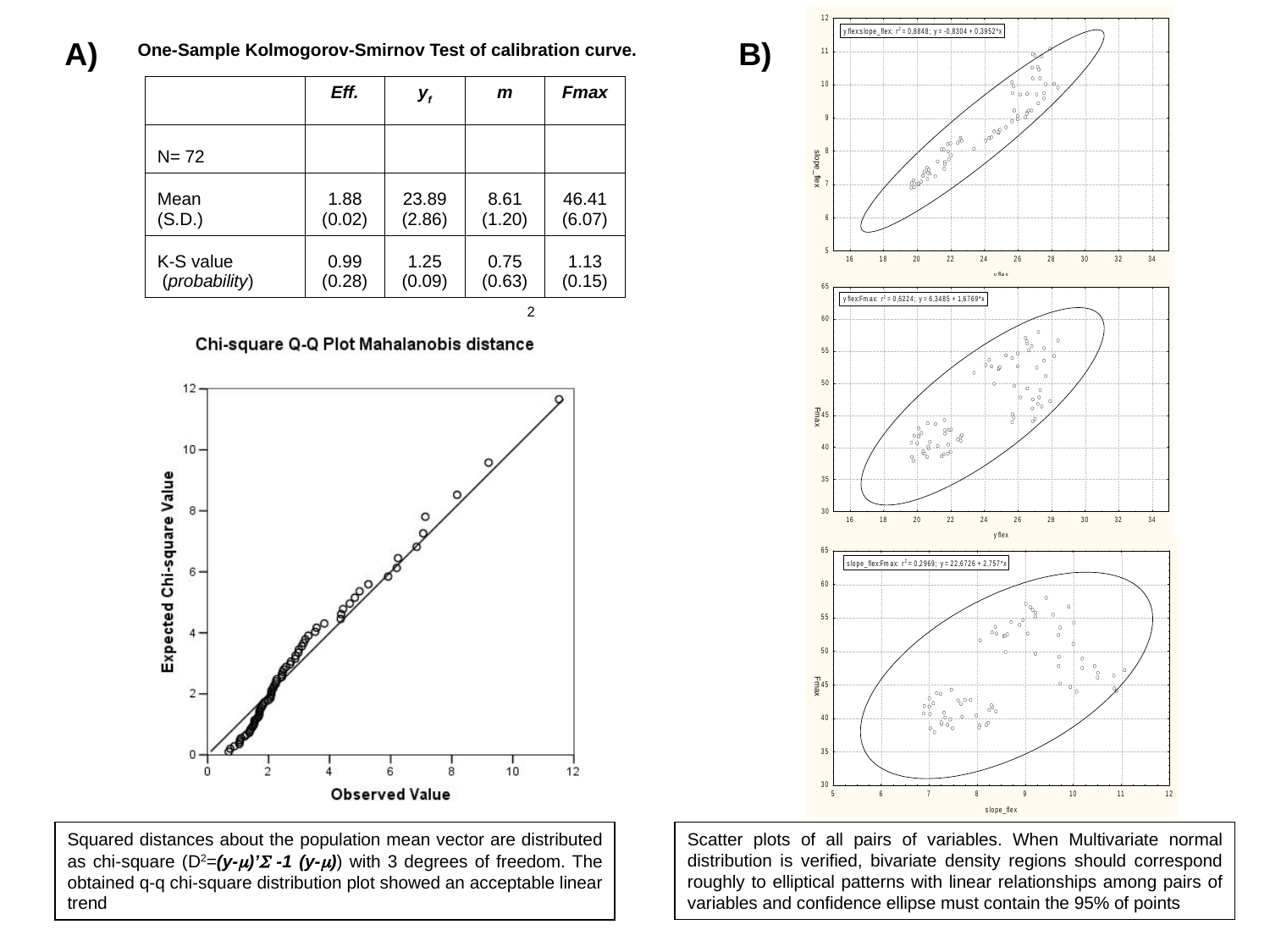

A)
B)
One-Sample Kolmogorov-Smirnov Test of calibration curve.
| | Eff. | yf | m | Fmax |
| --- | --- | --- | --- | --- |
| N= 72 | | | | |
| Mean (S.D.) | 1.88 (0.02) | 23.89 (2.86) | 8.61 (1.20) | 46.41 (6.07) |
| K-S value (probability) | 0.99 (0.28) | 1.25 (0.09) | 0.75 (0.63) | 1.13 (0.15) |
2
Scatter plots of all pairs of variables. When Multivariate normal distribution is verified, bivariate density regions should correspond roughly to elliptical patterns with linear relationships among pairs of variables and confidence ellipse must contain the 95% of points
Squared distances about the population mean vector are distributed as chi-square (D2=(y-μ)’ -1 (y-μ)) with 3 degrees of freedom. The obtained q-q chi-square distribution plot showed an acceptable linear trend
