# Supplementary material for: Shape based kinetic outlier detection in real-time PCR
Source: BMC Bioinformatics. 2010 Apr 12;11:186. doi: 10.1186/1471-2105-11-186 (PMC2873533; doi:10.1186/1471-2105-11-186)
Supplement: Additional file 4 — P-P plot of the variable Log(Nob/Nexp). [file 1471-2105-11-186-S4.PPT]

## Slide 1
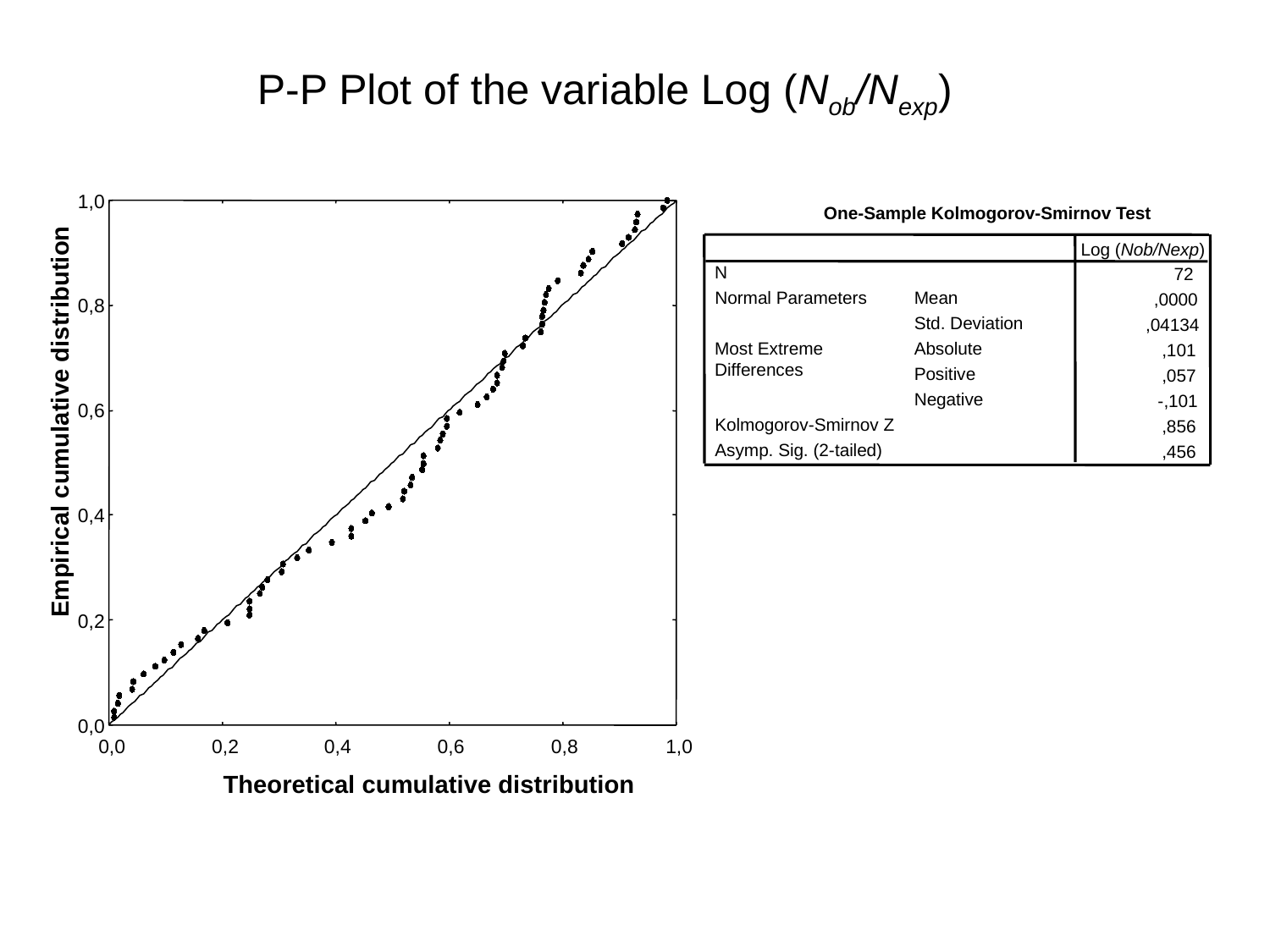

P-P Plot of the variable Log (Nob/Nexp)
1,0
One-Sample Kolmogorov-Smirnov Test
Log (Nob/Nexp)
N
72
Normal Parameters
Mean
,0000
0,8
Std. Deviation
,04134
Most Extreme
Absolute
,101
Differences
Positive
,057
Negative
-,101
0,6
Empirical cumulative distribution
Kolmogorov-Smirnov Z
,856
Asymp. Sig. (2-tailed)
,456
0,4
0,2
0,0
0,0
0,2
0,4
0,6
0,8
1,0
Theoretical cumulative distribution
